# Inhaled Dry Powder Antibiotics in Patients with Non-Cystic Fibrosis Bronchiectasis: Efficacy and Safety in a Real-Life Study

**DOI:** 10.3390/jcm9072317

**Published:** 2020-07-21

**Authors:** Miguel Ángel Martínez-García, Grace Oscullo, Esther Barreiro, Selene Cuenca, Angela Cervera, Alicia Padilla-Galo, David de la Rosa, Annie Navarro, Rosa Giron, Francisco Carbonero, Maria Castro Otero, Francisco Casas

**Affiliations:** 1 Pneumology Department, Hospital Universitario y Politécnico La Fe, 46026 Valencia, Spain; graceoscullo@gmail.com; 2 Centro de Investigación en Red de Enfermedades Respiratorias (CIBERES), Instituto de Salud Carlos III, 28029 Madrid, Spain; ebarreiro@imim.es; 3 Pneumology Department, Hospital del Mar-IMIM., 08003 Barcelona, Spain; 4 Pneumology Department, Hospital General de Castellón, 12004 astelló, Spain; selenecp@gmail.com; 5 Pneumology Department, Hospital General Universitario de Valencia, 46026 Valencia, Spain; angecj71@gmail.com; 6 Pneumology Department, Hospital Costa del Sol, 29604 Marbella, Spain; aliciapadillagalo@gmail.com; 7 Pneumology Department, Hospital Universitario Santa Creu i Sant Pau, 08041 Barcelona, Spain; david.rosa23@gmail.com; 8 Pneumology Department, Hospital Universitario Mutua Terrassa, 08221 Barcelona, Spain; annieknavarro@yahoo.com; 9 Pneumology Department, Hospital Univesitario la Princesa, 28006 Madrid, Spain; rmgiron@gmail.com; 10 Pneumology Department, Hospital de Jerez, 11407 Cádiz, Spain; pacocarboneros@gmail.com; 11 Pneumology Department, Hospital Central de la Defensa Gomez Ulla, 28028 Madrid, Spain; mariacastrootero@gmail.com; 12 Pneumology Department, Hospital Universitario San Cecilio, 18016 Granada, Spain; franciscocasas@neumosur.net

**Keywords:** dry-powder antibiotics, bronchiectasis, pseudomonas aeruginosa, inhaled antibiotics, colistin, tobramycin

## Abstract

Background: Nebulised antibiotics are habitually used in patients with bronchiectasis, but the use of dry powder inhaled antibiotics (DPIA) in these patients is extremely limited. This study seeks to analyse the efficacy and safety of DPIA in bronchiectasis patients. Material and methods: Multi-centre study of historic cohorts. All the hospital centres in Spain were contacted in order to collect data on patients with a diagnosis of bronchiectasis who had taken at least one dose of DPIA. Its efficacy was analysed in clinical, functional and microbiological terms by comparing the year before and the year after the prescription of DPIA. Adverse effects and variables associated with these effects, or any need to withdraw the drug, were also analysed. Results: 164 patients from 33 Spanish centres were included; 86% and 14% of these were treated with dry powder colistin and tobramycin, respectively. Chronic bronchial infection by *Pseudomonas aeruginosa* was present in 86% of these patients, and DPIA significantly reduced the number of exacerbations, the quantity and purulence of sputum and the isolation of pathogenic microorganisms. The most common adverse effect was cough (40%), particularly in cases of Chronic Obstructive Pulmonary Disease (COPD) and a previous cough and in those patients who had difficulties in handling the device. These factors were associated with a higher level of withdrawal of the treatment. There were no serious adverse effects. Conclusions: Our study suggests that DPIA are clinically efficacious and safe for treating bronchiectasis patients. Cough was shown to be the most common side-effect and reason for withdrawal of the treatment.

## 1. Introduction

Inhaled antibiotics have become strong contenders for use in patients with chronic bronchial infection (CBI), as in the cases of cystic fibrosis (CF) and bronchiectasis (BE) [1,2]. Although there is no official therapeutic indication for their use in BE, most experts in the field agree on the positive effects of this treatment in BE patients, and so its use is now recommended by international clinical guidelines in certain situations [3,4,5].

In recent years, several devices and preparations have been specifically developed for the administration of various antibiotics via inhalation. One of the most recently developed forms of administration is dry powder, used for products such as ciprofloxacin [6], vancomycin [7], tobramycin [8] and colistin [9]. Although the advantages of this form of administration seem obvious (smaller, more transportable devices that are easier to use and clean; faster administration; greater familiarity owing to the similarity with devices used for inhaled bronchodilators and corticosteroids; and a greater pulmonary deposition), some authors have suggested that it can produce more local adverse effects, such as cough, pharyngeal irritation, dyspnoea and haemoptysis [10]. Most of these side-effects are transitory, but nevertheless, they can result in withdrawal of the treatment and its replacement by an alternative one in some patients [11].

Although the efficacy and safety of nebulised antibiotics have been analysed in various studies, very little information is available about the efficacy and safety of dry powder antibiotics (DPIA) in patients with BE. However, dry powder inhalation, including antibiotics, might have clear advantages in the COVID pandemic era since no nebulization technique is needed. Accordingly, the objectives of our study were: (1) to identify the efficacy and safety of the treatment of BE patients with colistin and tobramycin in a dry powder formulation (these are the only two such drugs currently on the market) and (2) to identify the patient profile most susceptible to adverse effects and withdrawal of the treatment.

## 2. Experimental Section

### 2.1. Design

Multi-centre study of historic cohorts of patients with non-CF BE.

### 2.2. Patients

Patients aged over 18 years with a diagnosis of BE by chest high-resolution computerised tomography were included. The administration of at least one dose of an inhaled antibiotic, regardless of the indication for which it was described, was established as the criterion for inclusion. There were no criteria for exclusion due to the intention to perform a real-life study that would optimise the clinical applicability of the results obtained.

### 2.3. Methodology

The directory of the Spanish Society of Pneumology and Thoracic Surgery (SEPAR) was used to contact by e-mail all the hospital centres assigned to the Spanish National Health Service (787 hospitals). Those centres who agreed to participate in the study were sent a data collection form that would be filled in for each patient, with subsequent inclusion of these data on an SPSS or Excel database for further analysis. The patients formed part of the RIBRON registry (Spanish Computerised Bronchiectasis Registry), with the approval of the Ethics Committee (number: 001-2012. Hospital Josep Trueta, Girona). The prevailing legislation on the confidentiality of data was strictly adhered to. The information on the RIBRON [2] provided the patients′ general data and the data referring to the use of DPIA, which were also confirmed via the databases of the various hospital pharmacy departments. The study was approved by the University and Polytechnic La Fe Hospital Ethics Committee. Registry number: FPNT-CEIB-0415. All the patients signed the inform consent document to participate in the study.

### 2.4. Variables

All the information related to the patients′ baseline variables (general, anthropometric, clinical, etiological, functional, microbiological, radiological and evolutive) were collected via a standardised protocol pertaining to the national registry of bronchiectasis. The variables related to all the treatments prescribed, particularly DPIA (beginning and end dates of therapy, dose, type, adverse effects, reason for withdrawal, clinical and microbiological effectiveness) were similarly recorded. Patients and healthcare staff were given a questionnaire to evaluate whether patients had received instructions about using the inhalation device and information about possible adverse effects and how to handle them. Microbiological data and the number of exacerbations in both the year prior to and the year subsequent to the start of treatment with DPIA were also recorded. A semiquantitative assessment of microbiological cultures was made, being considered as positive when ≥10^3^ CFU were found. Chronic bronchial infection (CBI) was defined in accordance with the prevailing Spanish guidelines for bronchiectasis [12].

### 2.5. Statistical Analysis

All the variables were tabulated on the basis of their distribution. A Student T test for paired variables or chi square test was used to compare the clinical variables (particularly the number of exacerbations and the quantity and characteristics of sputum) and microbiological variables (particularly the number of isolations) recorded one year before and one year after the start of treatment with DPIA. Those patients with adverse effects (particularly those derived from the administration of the drug) were compared with those with no adverse effects, and those who were withdrawn from the treatment were compared with those who continued until the end of the study. In both cases, a Student T test or chi square test was used, depending on the distribution and characteristics of the variables. Finally, a logistic regression was used to analyse the variables independently associated with the presence of adverse effects (mainly cough) or the need to withdraw the treatment.

## 3. Results

**Figure 1:**
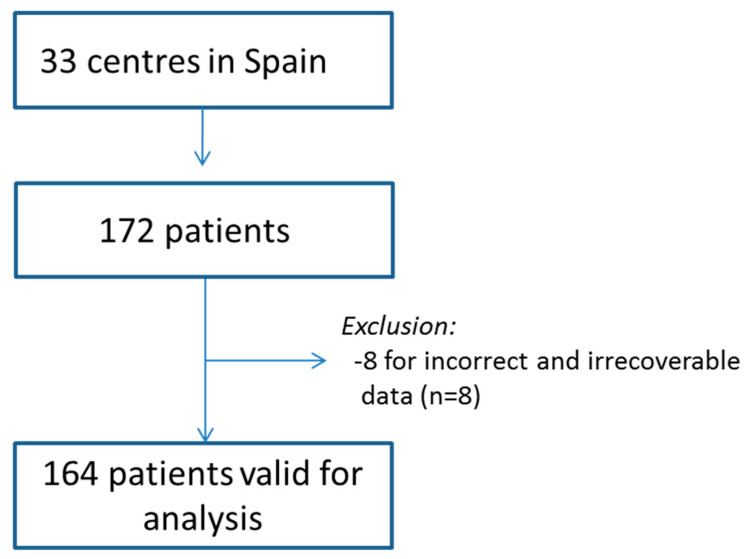
Flow chart of the study.

In the end, 33 centres in Spain participated in the study, with 172 patients, of whom 8 were not valid (Figure 1). Of the 164 patients validated for the analysis, 141 (86%) were treated with colistin and 23 (14%) with tobramycin. The patients′ general characteristics are presented in Table 1. The mean age was 65.7 (SD: 14.4) years and 47% were men; 33% of the patients presented a post-infectious aetiology and 26% were idiopathic. An association with Chronic Obstructive Pulmonary Disease (COPD) and asthma was observed in 37% and 20.6% of the patients, respectively. The mean forced expiratory volume during the first second (FEV1) (% pred) was 52.2%. CBI by *Pseudomonas aeruginosa* (PA) was present in 86% of cases. As regards treatment, 65.1% had been treated with inhaled antibiotics before, and 94.3% and 61.4% were taking bronchodilators and inhaled steroids, respectively, while 63% were also taking macrolides (Table 2). The mean FACED/EFACED were 3.7 (1.6) and 4.7 (2.1), respectively.

Table 3 presents factors related to treatment with DPIA. The mean treatment time was 6 months (SD: 6.5), ranging from 1 day to 30 months. The most common reason for the prescription was CBI by PA (86% of the patients). The first dose of the antibiotic was administered in a hospital in 81.1% of the cases, and the vast majority of the centres declared that they had informed their patients about possible adverse effects (particularly cough) and how to handle them. At least one adverse effect was observed in 54.2% of the patients, and 24.4% had to interrupt their treatment as a result of an adverse effect (cough in 84% of these cases).

### 3.1. Efficacy Analysis

**Figure 2:**
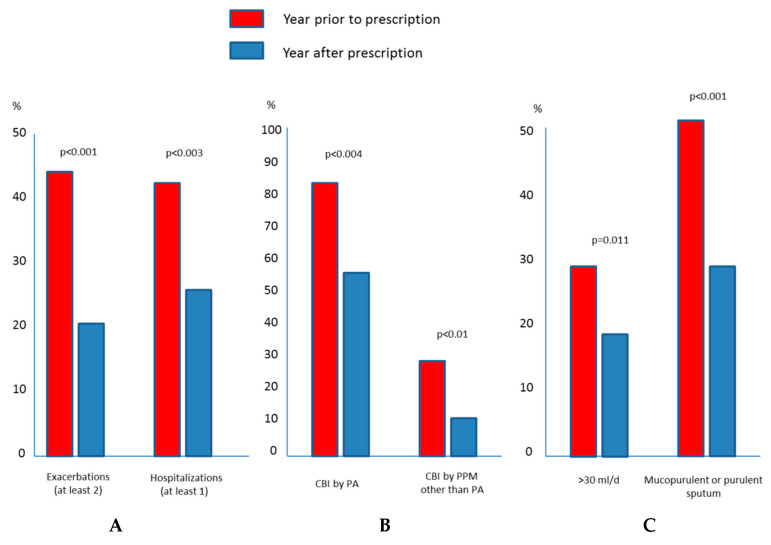
Clinical efficacy of dry powder antibiotics with respect to (**A**) exacerbations; (**B**) chronic bronchial infection and; (**C**) sputum characteristics.

There was a significant reduction in the number of exacerbations, both non-severe (1.9 (1.9) vs. 1.77 (1.9); *p* = 0.023) and severe (0.73 (1.2) vs. 0.33 (0.7); *p* < 0.001), in the comparison of the year before and after the start of treatment with a DPIA. There was a similar reduction in the percentage of patients defined as exacerbators, i.e., with at least two exacerbations or at least one hospitalization in the previous year (45% vs. 20%; *p* < 0.001; Figure 2).

From a microbiological viewpoint, the percentage of patients with CBI dropped significantly from 81% to 52% in the case of PA (*p* < 0.001), and from 29.4% to 10% in the case of other potentially pathogenic microorganisms (*p* < 0.001). No changes were found in the percentage of patients with fungi and non-tuberculous mycobacteria isolations 18.2% vs. 16.3%; *p* = 0.84 and 7.4% vs. 6.2%, *p* = 0.49, respectively.

There was a similar decrease in the percentage of patients producing >30 mL/d of sputum (31.6% vs. 19%; *p* = 0.01) or chronic muco-purulent or purulent sputum (55% vs. 29%; *p* < 0.001). No significant differences were observed, however, in the dyspnoea severity or lung function impairment (dyspnoea 1.5 (1.1) vs. 1.48 (1.2); *p* = 0.67) and FEV1 (% pred) (52.2 (8.9%) vs. 53.8 (8.7%)); *p* = 0.38. There were no statistically significant differences between the efficacy of colistin and tobramycin (Table 4).

### 3.2. Safety Analysis

Table 5 presents the patients who reported cough (or increased coughing subsequent to the prescription of DPIA) from the point at which they began the treatment. These patients were characterised by a greater age, more time since the diagnosis, a higher proportion of COPD diagnosis, more previous coughing, a higher number of previous exacerbations, greater difficulty in handling the device and a greater previous intake of an antibiotic. Table 5 shows how, of all these variables, the ones associated with a higher risk of cough as a side-effect were previous intake of an inhaled antibiotic (OR: 3.5), previous COPD (OR: 2.6), difficulty in handling the device, as perceived by the patient, (OR: 2.6) and a previous cough (OR: 2.9) (Table 6). These associations were independent of bronchiectasis severity (according to the FACED index value), age, gender and previous treatments, although it is the case that most of them were received previous bronchodilator treatment. No differences were observed between the patients who experienced cough and those without cough related to the type of DPIA antibiotic (85.1% of patients who experienced cough were under colistin treatment versus 86.6% who not experienced cough; *p* = NS).

### 3.3. Withdrawal of Treatment

Similarly, Table 7 shows that 24.4% of the patients were withdrawn from the treatment, 84% of these due to cough. The patients who were withdrawn presented more severe BE, more times since the onset of the symptoms, more previous coughing, greater difficulty in handling the device, less frequent administration of the first dose in a hospital and no instructions about use of the device. Of all these variables, the first dose administered outside a hospital (OR: 1.7), previous COPD (OR: 2.3), lack of instruction about the device (OR: 3.8) and previous coughing (OR: 2.7) were independently associated factors. There was no relationship between withdrawal from the treatment and age, gender or previous treatments (Table 8).

Those patients who received colistin presented a lower proportion of withdrawals from treatment (20 vs. 27%; *p* = 0.042), less difficulty in using the device and a smaller proportion of resistances to PA (3% vs. 22%; *p*: 0.001) versus those patients under tobramycin treatment.

## 4. Discussion

According to our results, DPIA is prescribed primarily to patients with CBI by PA who have already taken a nebulised inhaled antibiotic (most commonly colistin). This treatment seems to be effective in reducing the number and severity of the exacerbations, the purulence and quantity of sputum and the microbiological load. Cough is shown to be the most frequent adverse effect, and it is associated with patients with previous coughing or COPD, as well as inadequate handling of the device. The drug had to be withdrawn in around a quarter of cases in our study, usually on account of a persistent cough.

In the last years, some randomized controlled trials have been performed on the effect and safety of inhaled/nebulised antibiotics in patients with bronchiectasis with controversial results probably due to the different population selected and the methodological particularities of each trial [1]. Two of these studies were focused on the efficacy and safety of dry powder ciprofloxacin [13,14]. However only one of these studies showed positive results [13] in terms of clinical efficacy, and therefore, this drug was not approved by the regulatory agencies to be used in clinical practice in bronchiectasis. However, studies conducted with dry powder colistin and tobramycin on patients with CF have demonstrated that their efficacy is not inferior to that of nebulised antibiotics and that they are simpler to use (which could improve compliance) but also that they produce more adverse effects, usually cough, which is generally transitory but can represent a reason for withdrawing the medication [15,16]. In order to avoid this circumstance as far as possible, it is vital to instruct patients about both the appropriate use of the inhaler and the management of cough, if it should appear. Their efficacy and reasonable safety led to the arrival of DPIA (colistin and tobramycin) on the market to be used in patients with CF and the possibility to be used in other similar diseases such as bronchiectasis.

Patients with BE of a different origin (i.e., without CF) present different characteristics. The mean age is lower in CF, and respiratory comorbidities are more common in patients with non-CF BE. Even the microbiological profile is often different [17]. As a result, it is not possible to extrapolate therapeutic results obtained by different drugs from one disease to another. For example, medications such as DNase [18,19] and inhaled aztreonam [20,21] have proved efficacious in patients with CF but have not done so in those with BE (where they can even be counterproductive).

Although our study is not a randomised clinical trial, it does show how both the efficacy and the safety of DPIA in BE patients seems to be similar to those observed in CF patients. There was thus a reduction, after their administration, in the number and severity of exacerbations and in the purulence and quantity of sputum. No changes were observed, however, in dyspnoea or lung function, in keeping once again with the tendencies found in CF patients.

Most DPIA are prescribed as a result of a CBI by PA, following the previous use and withdrawal of a nebulised antibiotic, or for patients with a severe underlying disease (almost 80% present a moderate or severe FACED score [22]).

As in the case of CF, the most common adverse effect was cough, which appeared in over 40% of patients and lasted around 3 weeks on average. This adverse effect also triggered over 80% of the drug′s withdrawals. The factors that influenced both the appearance of cough and the withdrawal of the drug were previous coughing, the presence of COPD and insufficient instruction for the patient about how to use the device and manage any coughing. This suggests that any prescription of these drugs must be complemented by close monitoring of patients with previous airflow obstruction due to COPD or usual cough, as already described for other dry powder inhaled products [10,11]. Furthermore, it is essential to instruct patients about correct use of the device and give them information about how to handle any coughing. This educational aspect appears to be crucial, as it has already been reported that technical shortcomings in the inhalation of dry powder products increase the pharyngeal deposition and, therefore, the probable emergence of cough or other adverse effects [23,24].

No differences were observed in our study in efficacy or safety as regards either gender or age. Neither were there any significant differences between colistin and tobramycin as regards clinical efficacy and safety, although reports from patients indicated that the device used with the colistin treatment was simpler to use. Furthermore, colistin was also associated with a lower number of withdrawals (probably linked to the number of doses required, which is significantly lower than in the case of tobramycin). Finally, it was also observed that colistin presented a significantly lower rate of resistance to PA—a finding corroborated by previous studies [25].

The study has some strengths such us being the first real-life study on this topic with a wide and representative sample of our country. However, it has some limitations. The main limitation is that it is not a randomized clinical trial (that means that there was no arm of patients without DPIA), although its positive results do strengthen the assessment of the effect and safety of DPIA (especially colistin and tobramycin) in non-cystic bronchiectasis patients to confirm our findings and their clinical use in selected patients. Moreover, we have no data on other important clinical outcomes such us the effect on quality of life. Another limitation is that few patients were taking tobramycin, which means that the comparative results showed between colistin and tobramycin should be approached with caution.

## 5. Conclusions

In our study, the use of DPIA for bronchiectasis is most suited to patients with CBI by PA who have previously received a nebulised inhaled antibiotic. These drugs seem to be effective in reducing the number and severity of exacerbations, purulence, the quantity of sputum and the microbiological load. It was found that cough is the most frequent adverse effect, and this is particularly associated with patients with a previous cough or COPD and with patients handling the device inappropriately due to a lack of instruction and information about possible adverse effects. The drug had to be withdrawn in a quarter inadequate of cases in our study, usually on account of a persistent cough.

## Figures and Tables

**Table 1:** Baseline characteristics of the patients.

Baseline Characteristics (*n* = 164)	Mean (SD) or %
Age at prescription of IA, years	65.7 (14.4)
Gender Male/Female	47%/53%
BMI, kg/m^2^	24.5 (4.7)
Years since diagnosis of BE	12.8 (11.4)
Post-infectious aetiology	33%
Idiopathic	26%
Associated COPD	37%
Associated asthma	20.6%
Active smoker	3.8%
Accumulated packs/year	17 (32.4)
Dyspnoea (mMRC)	1.5 (1.1)
Cough prior to prescription of IA	83.8%
Common mucopurulent or purulent sputum	57.5%
Quantity > 30 mL/day	31.6%
FEV1, % pred	52.2 (20.7)
Number of lobes affected	2.99 (1.4)
Presence of cystic BE	28.7%
CBI by PA	86%
CBI by other PPM	29.4%
FACED score	3.7 (1.6)
Mild BE (0–2 points)	21.2%
Moderate BE (3–4 points)	50.7%
Severe BE (5–7 points)	28.1%
E-FACED score	4.7 (2.1)
Isolation of fungi	18.2%
Isolation of NTM	7.4%
Nº exacerbations in the year prior to the prescription of IA	1.9 (1.9)
Nº hospitalisations in the year prior to the prescription of IA	0.73 (1.25)

IA: Inhaled antibiotics; BMI: Body Mass Index; BE: bronchiectasis; COPD: Chronic Obstructive Pulmonary Disease; mMRC: Modified Medical Research Council; FEV1: Forced expiratory volume in 1 s; CBI: Chronic Bronchial Infection; PPM: Potentially Pathogenic Microorganism; FACED: (F) FEV1, (A) Age (C) Chronic colonization, (E) Extension, (D) Dyspnoea; E-FACED: FACED plus exacerbations; NTM: Non-tuberculous mycobacterium; IA: Inhaled antibiotics; PPM: Potentially Pathogenic Microorganism; CBI: Chronic Bronchial Infection.

**Table 2:** Baseline and previous treatments of the patients.

Treatments	%
Previous inhaled antibiotics	65.1%
Bronchodilators	94.3%
Inhaled corticosteroids	61.4%
Respiratory physiotherapy	70.3%
Mucolytic	41%
Hypertonic saline	17.9%
Macrolides	63%

**Table 3:** Characteristics associated with the use of dry powder inhaled antibiotics.

Variables	Mean (SD) or %
Use of dry powder colistin/tobramycin	86%/14%
Treatment time, months	6 (6.5) months, range: 1 day–30 months
Reason for prescription	
CBI by PA	86%
Intolerance of other IA	4.4%
Problem with handling other IA	2.4%
Need for portable IA	1.8%
First infection by PA	1.8%
Resistance to other IA	1.8%
CBI by other PPM (not PA)	1.8%
Information for the patient	
Handling of the inhaler	94.5%
Possible appearance of cough	93.2%
Handling of cough	87.2%
First dose in a hospital	81.1%
Difficulty in handling by the patient	25.6%
Adverse effects	
Cough	40.8%
Duration of cough	21.8 (44); range: 1–280 days
Dyspnoea	26.8%
Discomfort in the chest	15.8%
Haemoptysis	3.6%
Bad taste	6.1%
Appearance of at least 1 adverse effect	54.2%
Withdrawal due to adverse effects	24.4% (84% for cough)

CBI: Chronic Bronchial Infection; PA: *Pseudomonas aeruginosa;* IA: Inhaled antibiotics; PPM: Potentially pathogenic microorganisms.

**Table 4:** Dry powder inhaled antibiotics (DPIA) Efficacy in patients under colistin and tobramycin treatment.

Variable	Colistin Dry Powder 141 (86%)	Tobramycin Dry Powder 23 (14%)	Intergroup *p*-Value
Year Prior vs. Year After Prescription			
Non-severe exacerbations	2.01 (2.1) vs. 1.8 (2.1)	2.3 (2.5) vs. 1.7 (2.2)	NS
Severe exacerbations,	0.77 (1.3) vs. 0.43 (0.8)	0.61 (1.9) vs. 0.26 (2.2)	NS
Exacerbators	46% vs. 25%	59% vs. 21%	NS
CBI by PA	80% vs. 55%	85% vs. 50%	NS
CBI by PPM other than PA	20% vs. 11%	15% vs. 5%	NS
Sputum production > 30 mL/d	32% vs. 20%	33% vs. 15%	NS
Mucopurulent or purulent sputum	57% vs. 26%	56% vs. 17%	NS
Dyspnoea	1.5 (1.3) vs. 1.5 (1.4)	1.6 (1.5) vs. 1.7 (1.8)	NS
FEV1 (% pred)	53 (9.9%) vs. 55 (10.1%)	57 (14.2%) vs. 51 (14.1%)	NS

CBI: Chronic Bronchial Infection; PA: *Pseudomonas aeruginosa;* PPM: Potentially Pathogenic Microorganisms; NS: Non-significant.

**Table 5:** Appearance of cough as an adverse effect.

Variable	Appearance of Cough (40.8%)	Non-Appearance of Cough (59.2%)	*p*
Type of antibiotic (% colistin)	85.1%	86.6%	NS
Age	68.1 (15.8)	62.2 (12.9)	0.01
Gender (% women)	52%	53%	NS
FACED score	3.8 (1.5)	3.7 (1.7)	NS
Time since diagnosis	16.1 (13.2)	11.3 (9.5)	0.01
Associated COPD	43.9%	27.9%	0.004
Associated asthma	21%	20%	NS
Previous cough	92%	63%	0.001
Previous exacerbations	2.5 (2.2)	1.5 (1.5)	0.004
Difficulty in handling	32%	16%	0.001
CBI by PA	89%	83%	NS
Bronchodilators	89%	97%	NS
Previous use of inhaled antibiotics	77%	54%	0.002
Inhaled corticosteroids	59%	63%	NS
Hypertonic saline	22%	13%	0.09

FACED: (F) FEV1, (A) Age, (C) Chronic colonization, (E) Extension, (D) Dyspnoea; COPD: Chronic obstructive pulmonary disease; CBI: Chronic Bronchial Infection; PA: *Pseudomonas aeruginosa*; NS: Non-significant.

**Table 6:** Logistical regression. Factors associated with the appearance of cough after the start of dry powder antibiotic treatment.

Variable	OR (CI95%)	*p*
Previous use of inhaled antibiotic	3.5 (1.3–7.4)	0.003
COPD	2.6 (1.1–3.7)	0.002
Difficult in handling, according to the patient	2.6 (1.5–2.9)	0.002
Previous cough	2.9 (1.6–4.1)	0.034

COPD: Chronic obstructive pulmonary disease; OR: Odds Ratio; CI: Confidence Interval.

**Table 7:** Withdrawal of treatment with dry powder antibiotics.

Variable	Withdrawal of Treatment (40.8%)	No withdrawal of Treatment (59.2%)	*p*
Type of antibiotic (% colistin)	72.5%	90.3%	0.005
Age, years	66.5 (14.5)	63.4 (14.5)	NS
Gender (% women)	47%	55%	NS
FACED score	4.3 (1.5)	3.5 (1.6)	0.009
Time since diagnosis	16.1 (14)	12.3 (10.2)	0.01
Associated COPD	45%	26%	0.002
Associated asthma	22%	20%	NS
Previous cough	90%	65%	0.001
Previous exacerbations	2.1 (2.1)	1.9 (1.8)	NS
Difficulty in handling	27%	19%	0.03
No instruction on handling of cough	75%	94%	0.001
First dose in a hospital	71%	89%	0.001
CBI by PA	95%	83%	NS
Bronchodilators	92%	94%	NS
Previous use of inhaled antibiotics	69%	63%	NS
Inhaled corticosteroids	69%	59%	NS
Hypertonic saline	16%	18%	NS

FACED: (F) FEV1, (A) Age, (C) Chronic colonization, (E) Extension, (D) Dyspnoea; COPD: Chronic obstructive pulmonary disease; CBI: Chronic Bronchial Infection; PA: *Pseudomonas aeruginosa*; NS: Non-significant

**Table 8:** Logistical regression. Factors associated with the withdrawal of dry powder antibiotic treatment.

Variable	OR (CI95%)	*p*
First dose out of a hospital	1.7 (1.1–5.4)	0.023
COPD	2.3 (1.1–3.8)	0.005
Lack of instruction on handling cough	3.8 (1.9–4.9)	0.001
Previous cough	2.7 (1.4–4.3)	0.041

COPD: Chronic obstructive pulmonary disease. OR: Odds Ratio; CI: Confidence Interval.
